# Protease XIV abolishes NHE inhibition by empagliflozin in cardiac cells

**DOI:** 10.3389/fphys.2023.1179131

**Published:** 2023-07-26

**Authors:** Sha Chen, Cees A. Schumacher, Shirley C. M. Van Amersfoorth, Jan W. T. Fiolet, Antonius Baartscheer, Marieke W. Veldkamp, Ruben Coronel, Coert J. Zuurbier

**Affiliations:** ^1^ Amsterdam UMC, Location AMC, Department of Anaesthesiology, Laboratory of Experimental Intensive Care and Anaesthesiology (L.E.I.C.A.), Amsterdam, Netherlands; ^2^ Amsterdam Cardiovascular Sciences, Atherosclerosis & Ischemic Syndromes, Amsterdam, Netherlands; ^3^ Amsterdam UMC, Location AMC, Department of Experimental Cardiology, Amsterdam, Netherlands; ^4^ Amsterdam Cardiovascular Sciences, Heart Failure and Arrhythmias, Amsterdam, Netherlands

**Keywords:** SGLT2, NHE, protease XIV, empagliflozin, cardiomyocyte

## Abstract

**Background:** SGLT2i directly inhibit the cardiac sodium-hydrogen exchanger-1 (NHE1) in isolated ventricular cardiomyocytes (CMs). However, other studies with SGLT2i have yielded conflicting results. This may be explained by methodological factors including cell isolation techniques, cell types and ambient pH. In this study, we tested whether the use of protease XIV (PXIV) may abrogate inhibition of SGLT2i on cardiac NHE1 activity in isolated rabbit CMs or rat cardiomyoblast cells (H9c2), in a pH dependent manner.

**Methods:** Rabbit ventricular CMs were enzymatically isolated from Langendorff-perfused hearts during a 30-min perfusion period followed by a 25-min after-dissociation period, using a collagenase mixture without or with a low dose PXIV (0.009 mg/mL) present for different periods. Empagliflozin (EMPA) inhibition on NHE activity was then assessed at pH of 7.0, 7.2 and 7.4. In addition, effects of 10 min PXIV treatment were also evaluated in H9c2 cells for EMPA and cariporide NHE inhibition.

**Results:** EMPA reduced NHE activity in rabbit CMs that were not exposed to PXIV treatment or undergoing a 35-min PXIV treatment, independent of pH levels. However, when exposure time to PXIV was extended to 55 min, NHE inhibition by Empa was completely abolished at all three pH levels. In H9c2 cells, NHE inhibition by EMPA was evident in non-treated cells but lost after 10-min incubation with PXIV. NHE inhibition by cariporide was unaffected by PXIV.

**Conclusion:** The use of protease XIV in cardiac cell isolation procedures obliterates the inhibitory effects of SGLT2i on NHE1 activity in isolated cardiac cells, independent of pH.

## Introduction

Sodium-glucose cotransporter 2 inhibitors (SGLT2i) have revolutionized the medical treatment of diabetic and heart failure patients (Keller et al, 2022). Large clinical trials have demonstrated a significant decrease of approximately 30% in hospitalization for heart failure, and meta-analysis of different SGLT2i trials has also reported a substantial reduction in mortality (Cardoso et al, 2021). SGLT2i medications were originally developed to target the SGLT2 protein in the kidney, where it facilitates glucose reabsorption from the kidney filtrate into the blood. Inhibition of SGLT2 leads to glycosuria, resulting in the excretion of 40–75 g of glucose per day in diabetic or prediabetic patients (Veelen et al, 2023). Apart from their SGLT2-dependent effects on the kidney, including modulating of substrate metabolism, fluid balance, and hemodynamics, SGLT2i has been found to have direct effects on various cell types largely lacking SGLT2, such as cardiomyocytes, endothelial cells, platelets and fibroblast (Chen et al, 2022; Dyck et al, 2022). These effects also involve myocardial infarction and ischemia-reperfusion (I/R) (Andreadou et al, 2017). Indeed, it was shown that empagliflozin protection against infarct development in I/R was independent of the presence of SGLT2 (Chen et al, 2023). The sodium-hydrogen exchanger-1 (NHE1) was the first off-target cardiac mechanism of SGLT2i to be discovered (Baartscheer et al, 2017; Uthman et al, 2018). It has been demonstrated that SGLT2i inhibits NHE1 activity in isolated cardiomyocytes from rabbits and mice, leading to a decrease in intracellular Na^+^ and Ca^2+^ and an increase in mitochondrial calcium. While the inhibition of NHE1 by SGLT2i in isolated cardiomyocytes has been replicated in several studies involving different cell types (Spigoni et al, 2020; Jiang et al, 2022; Lin et al, 2022; Osaka et al, 2022; Peng et al, 2022; Uthman et al, 2022; Guo et al, 2023), other studies have reported conflicting results (Chung et al, 2021). In a previous investigation, we examined various experimental factors that differed among these conflicting studies, which could potentially account for the discrepancies.

It has been reported that enzymes used in cell isolation procedures can harm channel proteins embedded in the plasma membrane. For instance, protease XIV and XXIV, which are specific serine proteases, were found to degrade hERG1 potassium current and channel in HEK cells and cardiomyocytes (Rajamani et al, 2006). Consequently, the choice of cell isolation procedure can significantly impact the ion channels and exchangers within the plasma membrane. This raises the possibility that the loss of NHE1 inhibition by SGLT2i may be attributed to the use of specific enzymes in the cell isolation procedure.

We also questioned whether the PXIV effects on NHE1 by Empa was specific for freshly isolated rabbit cardiac cells or more a general phenomenon applicable to other cardiac cells as well. We therefore also used another cardiac cell type. Therefore, in this study, we aimed to investigate whether the exposure time of serine protease XIV disrupts inhibition of NHE1 by SGLT2i in freshly isolated rabbit cardiac cells. We next tested whether 2) this effect was dependent on pH, 3) was also present in cardiac precursor cells, and 4) NHE inhibition by the classic NHE inhibitor cariporide was also affected by PXIV. We conclude that inhibition of the NHE by EMPA is strongly dependent on duration of protease incubation both in rabbit cardiomyocytes and rat cardiac precursor cells, and that it is independent on pH.

## Methods

### Preparation of isolated ventricular cardiomyocytes by enzymatic dissociation

**FIGURE 1:**
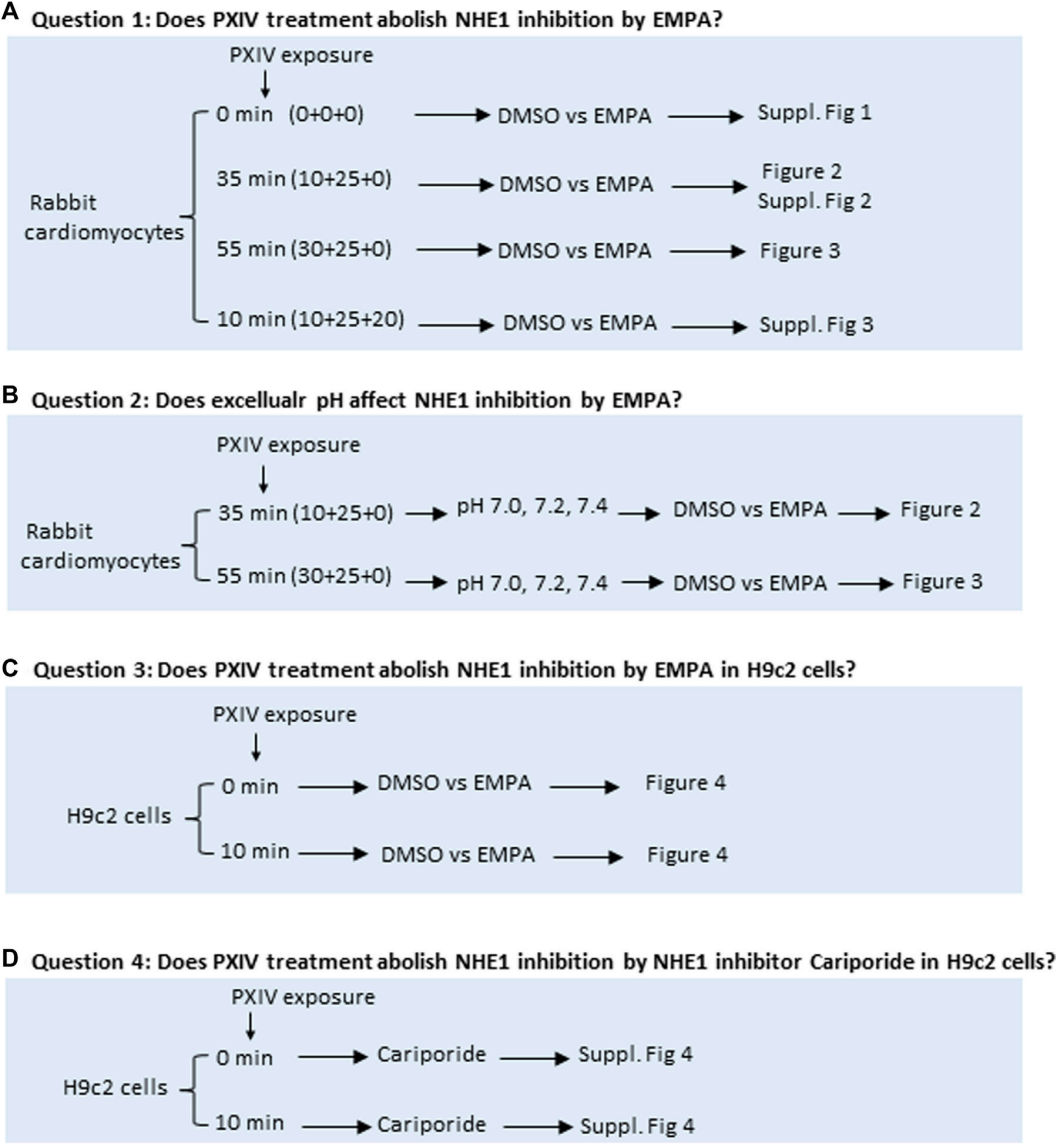
Overview of the four research questions addressed to study effects of various durations of Proteinase XIV exposure and extracellular pH’s for determination of NHE1 activity in isolated rabbit cardiomyocytes and H9c2 cells. **(A, B)** Rabbit cardiomyocytes are isolated on a Langendorff set-up by perfusing with a fixed mixture of collagenases with or without different treatment periods of proteinase XIV. Next, rabbit cardiomyocytes are incubated with the pH fluorescent probe SNARF-AM in a water bath for 20 min in absence or presence of PXIV. NHE1 activity in cardiomyocytes is measured by fluorescence microscopy where different pH conditions of HEPES solution treated with or without EMPA are applied during the measurement. **(C, D)** H9c2 cells were treated with DMSO, EMPA or NHE1 inhibitor Cariporide with or without PXIV during the NH4^+^-treatment and NHE1 activity measurements. EMPA: Empagliflozin. Fig 1A For question 1 and 2, exposure time of PXIV is given as the sum of (X+Y+Z), Where by X=PXIV exposure during cell dissociation on Langendorff system, Y=PXIV exposure during after-dissociation in gyrotory water bath shaker (cells are taken out from gyotory every 5 min), Z=PXIV exposure during SNARF-AM incubation in waterbath. For questions 3 and 4, PXIV exposure is during the 10min NH4^+^-treatment.

The study was approved by the local animal experiments committee of the Academic Medical Center, Amsterdam, Netherlands. Rabbits were housed for at least 1 week, with a 12 h day/night cycle, and food and drinking water *ad libitum*. Male New Zealand White rabbits (2.5–3.5 kg) were anaesthetized with 35 mg/kg ketamine and 10 mg/kg xylazine subcutaneously and heparinized with a bolus of 1000 IU heparin (intravenously). Subsequently, the animals were euthanized by pentobarbital 50 mg/kg intravenously, the thorax was opened and the heart was quickly removed and submerged in ice-cold perfusion solution (composition see below). After cannulation of the aorta, the heart was mounted on a Langendorff perfusion setup, and perfused for 15 min at constant pressure (50 mmHg) at 37°C with a modified Tyrode’s solution containing (in mM) 128 NaCl, 4.7 KCl, 1.45 CaCl_2_, 0.6 MgCl_2_, 27 NaHCO_3_, 0.4 NaH_2_PO_4_ and 11 glucose (pH maintained at 7.4 by equilibration with a mixture of 95% O_2_ and 5% CO_2_) to remove remaining blood from the heart. Next, perfusion was changed to a nominally calcium-free solution containing (in mM) 16.8 HEPES, 146.4 NaCl, 3.3 KHCO3, 1.4 KH_2_PO_4_, 1.0 NaHCO_3_, 2.0 MgCl_2_, 0.01 CaCl_2_, 11.0 glucose, pH 7.3 (NaOH). After 15 min, collagenase type B (0.15 mg/mL, Roche 11088815), collagenase type P (0.05 mg/mL, Roche 11213865), trypsine inhibitor (0.1 mg/mL, Roche 10109878), 0.2 mg/mL hyaluronidase (Sigma H-3506), and creatine (10 mM) were added, and the heart was perfused for another 30 min at a constant flow in a recirculating manner. During the final 30-min period, protease XIV (Sigma H-3506) was either absent, present only during the last 10 min of perfusion, or present throughout the entire 30-min perfusion. Subsequently, the left ventricular wall was further dissociated using the following after-dissociation protocol: the tissue was cut into small pieces, incubated in the enzyme-containing nominally calcium-free solution with or without PXIV (see Figure 1), and gently agitated using a gyrotory water bath shaker for 25 min. During the last 10 min, 1% albumin (fatty acid free, Roche 10775835001) was added to the enzyme-containing dissociation solution. All dissociation solutions were saturated with 100% O_2_ and the temperature was maintained at 37°C. Cells were allowed to sediment and were resuspended in enzyme-free dissociation solution supplemented with 1% albumin and 1.3 mM CaCl_2_. Only rod-shaped myocytes with clear striations were selected for NHE1 activity measurement.

### Cell culture

H9c2 rat cardiomyoblast cells were obtained from ATCC (American Type Culture Collection) and cultured in high glucose (4,500 mg/L) Dulbecco’s modified Eagle’s medium (DMEM) supplemented with 10% Fetal Calf Serum (FCS) and 100 U/ml of Penicillin/Streptomycin. In brief, H9c2 cells were digested with trypsin (0.25%), recovered by centrifugation (5 min, 1,000 rpm) and re-suspended in plating medium. Cells were grown in an atmosphere of 95% O_2_, 5% CO_2_ in a humidified incubator. Stock cultures were passaged at 2- to 3-day intervals. 50.000 cells were seeded and grown on coverslips for NHE1 activity measurement.

### NHE1 activity measurement

To measure intracellular pH, isolated rabbit cardiomyocytes and H9c2 cells were incubated with 10 μM SNARF-AM (Thermofisher, C1270) for 30 min in a 37 °C water bath (Figure 1A) in HEPES solution (mM): HEPES 17.0, NaHCO_3_ 1.0, KHCO_3_ 3.3, KH_2_PO_4_ 1.4, CaCl_2_ 1.3, MgCl_2_ 2.0, NaCl 144, pH adjusted with NaOH to 7.0 or 7.2 or 7.4. In a separate set of experiments, isolated rabbit cardiomyocytes which had been exposed to 0.009 mg/mL Protease XIV for 35 min during enzymatic dissociation protocol (Figure 1), were re-incubated with 0.009 mg/mL Protease XIV for an additional 20 min during SNARF-AM incubation. SNARF-loaded rabbit cardiomyocytes were attached to a poly-D-lysine (0.1 g/L) treated coverslip for NHE1 activity measurement. The general workflow of the procedures described above and the research questions addressed is depicted by a schematic drawing in Figure 1A, B. Experiments were conducted using a temperature controlled (37 °C) perfusion chamber (height 0.4 mm, diameter 10 mm, volume 30 μL) in which the coverslips were placed. NHE1 activity was measured by recording SNARF-fluorescence (580/640 nm emission; 515 nm excitation) following a NH_4_^+^ pulse (Baartscheer et al, 2017; Uthman et al, 2022). Shortly, after 30 s stabilization, medium was quickly replaced (<1 s) with the same solution now containing 20 mM NH_4_Cl for 10 min, resulting in intracellular alkalosis. After 10 min, the NH_4_Cl was quickly replaced (<1 s) with normal solution, which resulted in an almost instantaneously intracellular acidosis, followed by the recovery from acidosis, which was monitored for the next 5 min. The effect of 1 µM EMPA (MedChem Express, Monmouth Junction, NJ, USA) or 0.02% DMSO (Sigma/Merck, D2650) was tested on recovery of acidosis and these compounds were continuously present during the NH_4_Cl pulse and the washout period of NH_4_Cl. Additionally, H9c2 cells, were subjected or not to 0.009 mg/mL Protease XIV during the 10 min NH_4_Cl pulse and the washout period of NH_4_Cl during which either DMSO, EMPA or cariporide (10 μM) were also present, to address the questions whether NHE inhibition by EMPA or the classic NHE inhibitor cariporide was dependent on the presence of PXIV in this different cell type. (Figure 1C, D). The initial rate of intracellular H^+^ recovery following washout of ammonium is used as estimate of NHE1 activity. This initial rate equaled the slope of the linear fit of the intracellular H^+^ during the first 50 s of H^+^ recovery.

### Statistics

Statistical analysis was performed using GraphPad Prism v9. Results are presented as mean ± SEM for normally distributed data or median ± IQ for non-normally distributed data. Data normality was examined using the Shapiro-Wilk test (with *α* = 0.05). For normally distributed data, the Paired t-test (unpaired 2-tailed) was used for 2-group analysis. For non-normally distributed data, the Mann-Whitney test was used for 2-group analysis, and the Kruskal-Wallis test with Dunn’s multiple comparisons test was used for multiple-group analysis. ns *p* > 0.05, *p < 0.05, **p < 0.01, ***p < 0.001, ****p < 0.0001.

## Results

**FIGURE 2:**
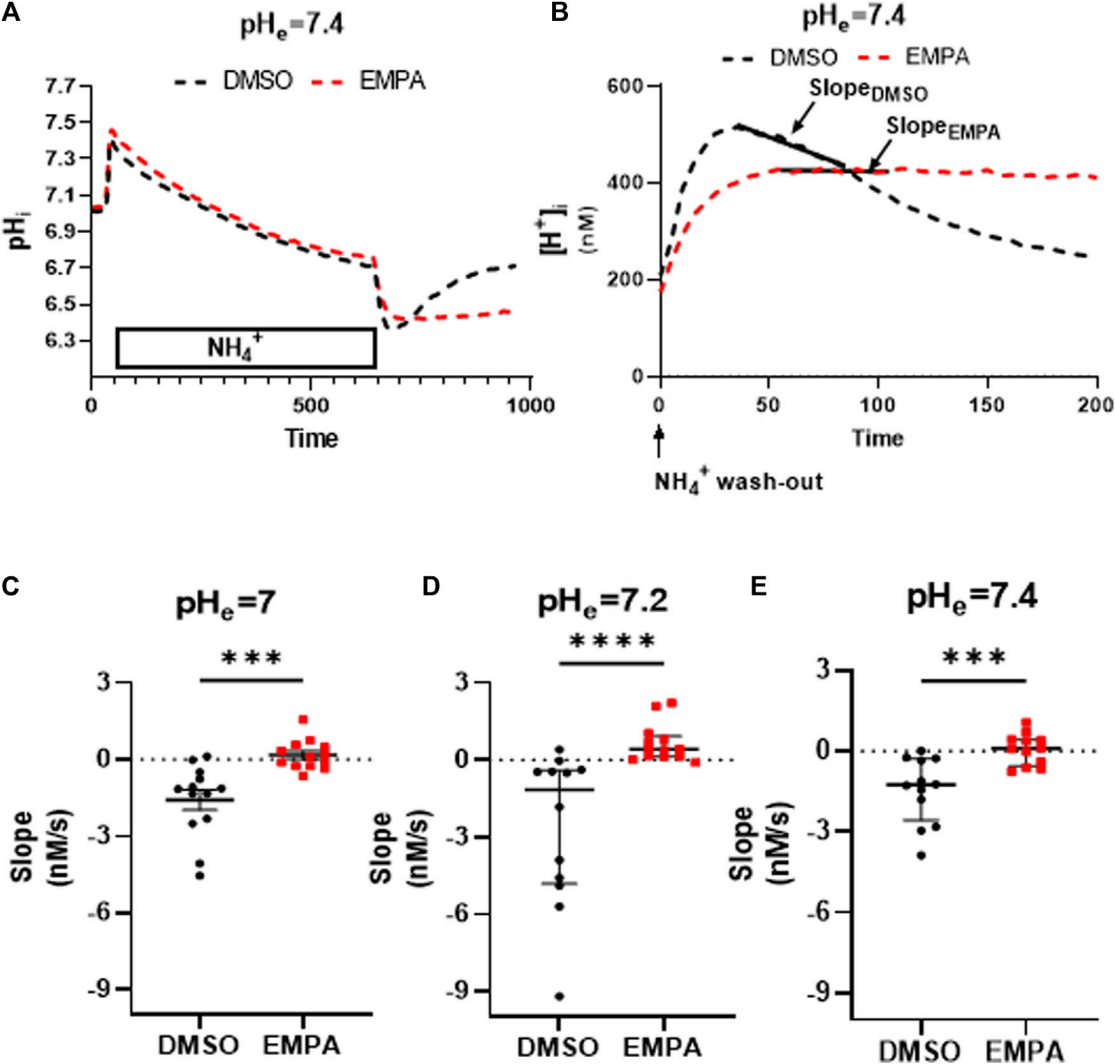
Empagliflozin inhibits NHE1 activity in rabbit CMs exposed to PXIV for 35 min during enzymatic dissociation. **(A)** Average curve changes of intracellular pH in response to a NH_4_+pulse for DMSO- and EMPA-treated CMs; **(B)** Average curve changes of intracellular [H^+^] during the first 200 s after NH_4_^+^ wash-out, showing the slope; **(C–E)** NHE1 activity indicated by the slope of the linearly fitted [H^+^] recovery for DMSO- and EMPA-treated cell at different extracellular pH’s (pH = 7.0: *n* = 13/13 cells from 7 rabbits, Paired t-test (two tailed); pH = 7.2: *n* = 12 (DMSO)/13(EMPA) cells from 7 rabbits, Mann-Whitney test; pH = 7.4: *n* = 12/12 cells from 7 rabbits, Paired t-test (two tailed)). EMPA: Empagliflozin. Slope (Δ[H^+^]/Δs) of the linear fit of the first 50 s of intracellular [H^+^] recovery. ***p < 0.001, ****p < 0.0001.

We first established the presence of NHE1 inhibition by EMPA with our standard cell isolation procedure without PXIV treatment. Typical examples from rabbit ventricular CMs clearly demonstrate the failure to recover from intracellular acidosis in the presence of EMPA compared to DMSO (Supplementary Figure S1A). NHE1 activity was calculated from the slope of the linear fit of the first 50 s of [H^+^] recovery after [H^+^] had reached its maximum value (Supplementary Figure S1B). At an extracellular pH of 7.4, EMPA significantly altered the negative slope of the linear fitted [H^+^] recovery of DMSO-treated cells towards a more positive value, indicating decreased recovery of [H^+^] due to NHE 1 inhibition by EMPA (Supplementary Figure S1C). We next investigated whether a 35 min exposure toPXIV (0.009 mg/mL) during the dissociation protocolaffected NHE1 inhibition by EMPA, and whether this inhibition was dependent on the extracellular pH. The mean pH- traces during and after the NH_4_^+^ treatment for DMSO- and EMPA-treated CMs are shown in Figure 2A, demonstrating strong inhibition of pH recovery following NH_4_^+^ withdrawal in the presence of EMPA as compared to DMSO only. The individual pH traces per animal for each of the 7 rabbits are given by Supplementary Figure S2. Following the conversion of these mean pH tracings into mean changes of [H^+^] (Figure 2B), the slope of the linear fit of [H^+^] recovery approximates zero (0.06 ± 0,57) in the presence of EMPA, whereas the slope of the linear fir is −1.44 (−1,44 ± 1,23, *p* < 0.001) in the presence of DMSO, indicative of inhibition of [H^+^] recovery by EMPA at pH 7.4. The individual and average slope values for 35 min PXIV at the different extracellular pH’s, are depicted in Figure 2C–E. At pH 7.0, 7.2 and 7.4 EMPA significantly reduced NHE1 activity as compared to DMSO treated rabbit cardiomyocytes (Figure 2C). This indicates that short term PXIV treatment or changes in extracellular pH in the range of 7.0–7.4, leave NHE1 inhibition by EMPA intact.

**FIGURE 3:**
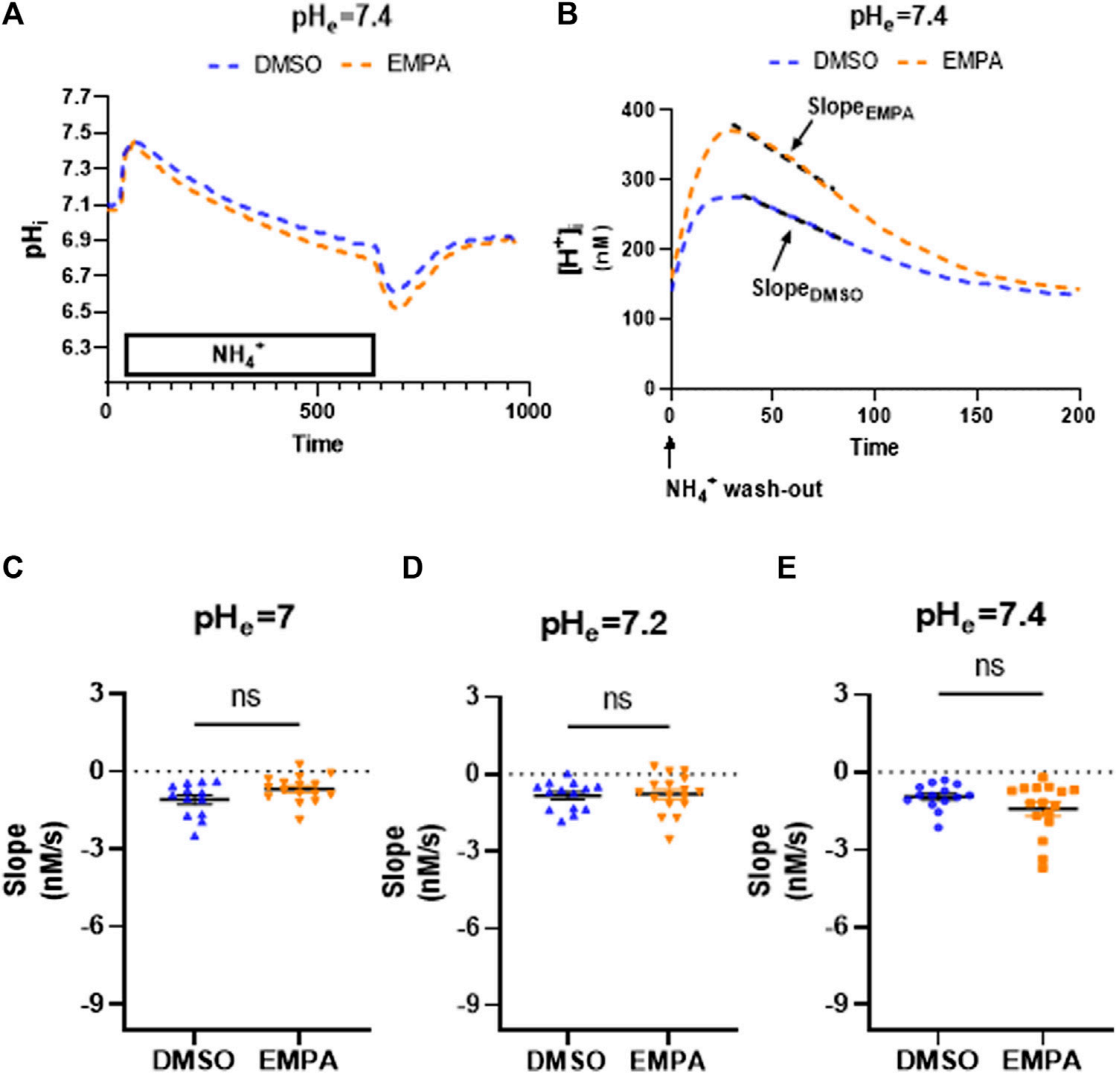
Empagliflozin does not inhibit NHE1 activity in rabbit CMs exposed to PXIV for 55 min during enzymatic dissociation. **(A)** Average curve changes of intracellular pH for DMSO and EMPA treatment; **(B)** Average curve changes of intracellular [H^+^] during the first 200 s after NH_4_^+^ wash-out, showing the slope for DMSO and EMPA. **(C–E)** NHE1 activity as reflected by the slope for DMSO- and EMPA-treated cell at different extracellular pH’s (pH = 7.0: *n* = 13/16 cells from 5 rabbits, Paired t-test (two tailed); pH = 7.2: *n* = 14(DMSO)/17(EMPA) cells from 5 rabbits, Paired t-test (two tailed); pH = 7.4: *n* = 14/16 cells from 6 rabbits, Paired t-test (two tailed)). EMPA: Empagliflozin. Slope (Δ[H^+^]/Δs) of the linear fit over first 50 s of intracellular [H^+^] recovery. ns *p* > 0.05.

We then investigated whether prolonged exposure to PXIV (55 min) during the enzymatic dissociation protocolaffects NHE1 inhibition by EMPA or influences pH effects on inhibition (Figure 3). The mean pH tracings following 55 minutes PXIV (Figure 3A), now show pH recovery to occur under both DMSO and EMPA conditions. Accordingly, the slope of the linear fit of the mean [H^+^] tracing for EMPA shows a significant negative slope, similar to the DMSO condition, indicative of pH recovery (Figure 3B). The absent of an EMPA-effect was consistent at extracellular pH 7.0, 7.2 and 7.4. Thus, the increased exposure time of PXIV during the dissociation procedure, abolished the effects on NHE activity by EMPA, independent of the extracellular pH levels (Figure 3C).

Next, we determined whether PXIV can also abrogate EMPA-NHE1 inhibition when prolonged exposure to PXIV is applied after the cell isolation procedure. CMs that were exposed for 35 min to PXIV during the enzymatic dissociation procedure, were subsequently incubated for another 20 min with PXIV during SNARF-incubation. Supplementary Figure S3A, B are examples of pH and [H^+^] changes in response to an NH_4_^+^ pulse and subsequent NH_4_^+^ washout in cells treated solely for 35 min with PXIV (extracellular pH of 7.2), showing a clear inhibitory effect of EMPA on NHE1 activity (Supplementary Figure S3C). On the other hand, CMs that were exposed to an additional 20 min of PXIV, EMPA was not effective in blocking NHE1 activity (Supplementary Figure S3D–F).

**FIGURE 4:**
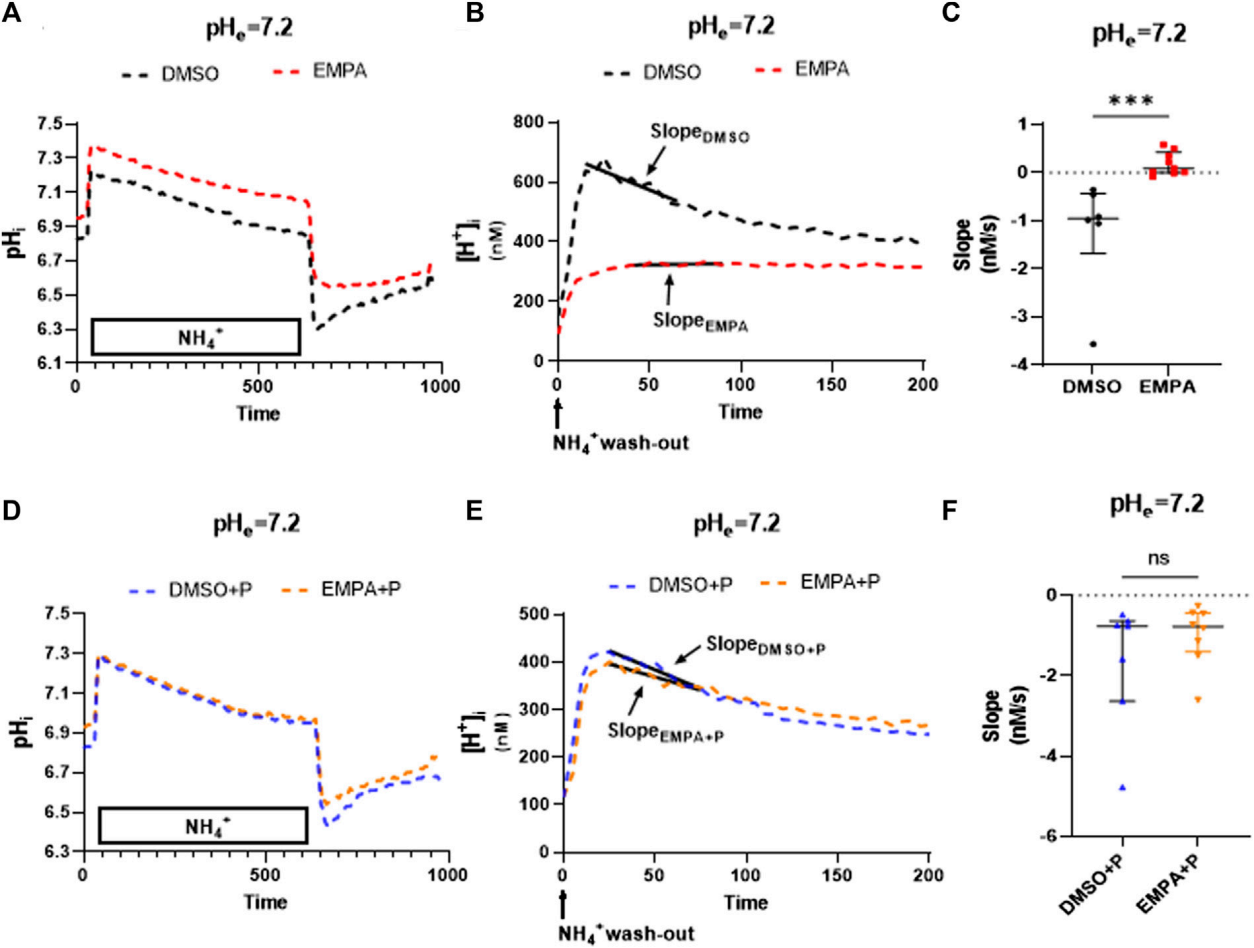
EMPA inhibits NHE1 activity in H9c2 cells, with loss of NHE1 inhibition after PXIV treatment. H9c2 cells not treated with PXIV *versus* H9c2 cells treated for 10 min with PXIV. **(A–C)** Average curve changes of intracellular pH **(A)**, [H^+^] and slope fitting **(B)** and summary data of slope **(C)** for H9c2 cells not treated with PXIV (n = 6(DMSO)/8(EMPA) cells from 7 independent experiments, Mann-Whitney test). **(D–F)** Average curve changes of intracellular pH **(D)**, [H^+^] and slope fitting**(E)** and summary data of NHE1 activity **(F)** for H9c2 cells treated with 10 min PXIV incubation during NH4^+^ pulse and the pH recovery period (*n* = 7/8 cells from 7 independent experiments, Mann-Whitney test). EMPA: Empagliflozin. P: PXIV. Slope (Δ[H^+^]/Δs) is the linear fit over first 50 s of intracellular [H^+^] recovery. ns *p* > 0.05, ***p < 0.001.

To study whether the PXIV effects also extend to other cell types, we employed the rat cultured myoblast cell line H9c2. EMPA effects on NHE activity at pH 7.2 were determined in the absence or presence of 0.009 mg/mL PXIV, administered simultaneously with the 10 min NH_4_^+^ treatment (Figure 4). Figure 4A, B show that EMPA also in this cardiac cell type inhibits the NHE1, as reflected by the mean course of changes in intracellular pH and H^+^ following the NH_4_^+^ pulse in the presence of EMPA (compared to DMSO-treatment), and the accompanying averaged and individual slope values (Figure 4C). However, when cells were treated with PXIV during the NH_4_^+^ pulse and the subsequent pH recovery period, recovery of pH (Figure 4D) and H^+^ (Figure 4E) in the presence of EMPA is present and similar to recovery with DMSO. Accordingly, the summarized slopes of H^+^ recovery were not different between EMPA and DMSO treated cells (Figure 4F).

Finally, we examined whether PXIV treatment also affects NHE1 inhibition by the classical inhibitor of NHE1, cariporide. H9C2 cells were subjected to 10 μM cariporide with or without PXIV treatment during and after the NH4^+^ pulse (Supplementary Figure S4). Supplementary Figure S4A–C clearly illustrates that NHE1 inhibition by cariporide is unaffected by PXIV.

## Discussion

The key findings of this study are as follows: 1) Protease XIV can completely abrogate the inhibition of NHE1 by empagliflozin in primary cardiomyocytes and cultured cardiomyoblast cells, and 2) Empagliflozin’s inhibition of NHE1 is independent of extracellular pH levels ranging from 7.0 to 7.4. These results highlight the significance of experimental conditions in generating reproducible data and emphasize the careful selection of enzymes used in cell isolation procedures tuned to the specific mechanism under investigation.

### Enzymes used for cardiac cell isolation procedures for NHE1 research

In 2017 we were the first to report the inhibition of NHE1 activity by empagliflozin in isolated cardiac cells from rabbit hearts (Baartscheer et al, 2017). In that study, cardiac cells were isolated by a 30-min perfusion of the isolated rabbit heart with collagenase B (15 mg/100 mL), collagenase P (5 mg/100 mL), hyaluronidase (20 mg/100 mL) and a trypsin inhibitor (10 mg/mL), followed by 25-min incubation of the cells with a similar enzyme solution. In a subsequent study, cells were obtained from isolated mouse hearts by perfusing with liberase TM (0.032 mg/mL) and elastase (1.6 U/mL) for a duration of 15 min (Uthman et al, 2018). A similar isolation procedure was used to obtain isolated mouse cardiomyocytes to explore the potential impact of insulin on NHE1 inhibition by empagliflozin (Uthman et al, 2019). Strong inhibition NHE1 activity by SGLT2i was observed in all these studies. Additionally, NHE1 inhibition by empagliflozin in isolated mouse cardiomyocytes was also observed by Trum and co-workers (Trum et al, 2020) employing liberase and trypsin as digestive enzymes, and by Peng and co-workers (Peng et al, 2022) employing trypsin and collagenase enzymes. In contrast, isolated rat cardiomyocytes obtained from perfusion of the isolated rat heart with protease VIX (0.025–0.09 mg/mL) and collagenase type II (0.9–1.0 mg/mL) did not show NHE1 inhibition by SGLT2i (Richards et al, 2020; Chung et al, 2021). The present work demonstrated that exposing rabbit cardiomyocytes during the enzymatic dissociation protocol for 55 min to a low dosage of PXIV (0.009 mg/mL), abolished the inhibitory effects of EMPA on NHE1. Similarly, a treatment of only 10 min with 0.009 mg/mL PXIV was sufficient to eliminate EMPA’s effects on NHE1 in H9c2 cells. Therefore, the use of PXIV may likely explain the absence of NHE1 inhibition in some studies involving isolated cardiomyocytes. It has been demonstrated that the protease XXIV selectively degrades the S5-pore extracellular linker of the KCNH2 (hERG1) protein, which is responsible for the rapidly activating delayed rectifier K^+^ current (Rajamani et al, 2006). Docking studies have suggested that SGLT2 inhibitors interact with the extracellular Na^+^-binding site of NHE. Further research is required to elucidate the precise molecular domain of the NHE protein that is affected by PXIV proteolysis. Specifically, it is important to determine whether the extracellular Na^+^-binding site or another extracellular region of NHE1 is affected. Notably, the inhibition of NHE1 activity by cariporide is not affected by PXIV treatment, indicating different molecular motif for NHE1 inhibition by cariporide *versus* SGLT2i. Even if the exact molecular mechanism(s) of the effect of proteases are unknown, this study emphasizes that study methods are key to the interpretation of the results. We speculate that our observations potentially are applicable to the function of other extracellular sarcolemma-bound proteins as well.

### Persistent NHE1 inhibition by EMPA at different extracellular pH levels

In the first reports demonstrating NHE1 inhibition by SGLT2 inhibitors, the extracellular pH during measurements at 37° was set at 7.2 (Baartscheer et al, 2017; Uthman et al, 2018), whereas this pH equaled 7.4 in reports showing absence of NHE1 inhibition by EMPA (Chung et al, 2021). A previous study indicated a potential decrease in NHE1 inhibition by EMPA at higher pH levels (Murphy and Eisner, 2021; Zuurbier et al, 2021). Because NHE1 activity is critically dependent on extracellular pH, with extracellular acidosis decreasing NHE1 activity (Vaughan-Jones and Wu, 1990; van Borren et al, 2004), we also changed pH during our studies (extracellular pH 7.0–7.4). The results clearly demonstrate that NHE1 inhibition was consistently present at all tested pH levels, with no decreased inhibition at higher extracellular pH.

In conclusion, robust inhibition of the NHE1 by EMPA was observed in non-PXIV treated rabbit cardiomyocytes and rat H9c2 cardiomyoblast cells. However, prolonged exposure to PXIV in cardiac cells abolished NHE1 inhibition by EMPA, highlighting the need for caution when using of PXIV in cardiac cell isolation procedures to examine the effects of SGLT2 inhibitor on cardiac NHE1 activity.

### Methodological considerations

Ideally, NHE activity is determined by exponential fitting of the [H^+^]_I_ decay curve over a full range of pHi’s, whereby the obtained decay rate constant λ then reflects NHE activity independent of the pH. However, with EMPA or Cariporide there often was no [H^+^]_I_ decay at all (resulting in an undefined decay constant), making exponential fitting strategies inappropriate. We therefore chose to use linear fitting of [H^+^]_I_ changes following the first 50 s after withdrawal of NH_4_^+^, with the slope of the linear fit as an index of NHE activity. Although there were sporadically differences in the surge of the [H^+^]_I_ between experiments resulting in a slightly different pH trajectories over which NHE activity was determined, most linear fits were similar for their pH trajectories over which the slope or NHE activity was determined. This justifies the use of slopes of linear fits as index of NHE activity as a measure of NHE activity.

## Data Availability

The original contributions presented in the study are included in the article/Supplementary Material, further inquiries can be directed to the corresponding author.
