# Supplementary material for: Protease XIV abolishes NHE inhibition by empagliflozin in cardiac cells
Source: Front Physiol. 2023 Jul 26;14:1179131. doi: 10.3389/fphys.2023.1179131 (PMC10410854; doi:10.3389/fphys.2023.1179131)
Supplement: Supplementary file 1 [file DataSheet1.PDF]

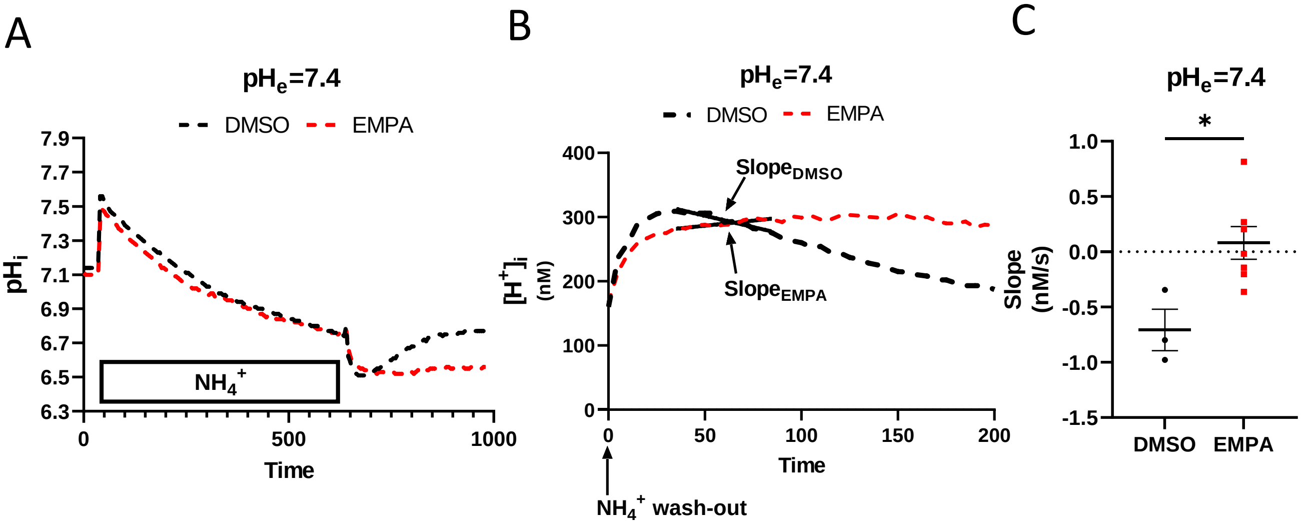

Supplementary figure 1. Empagliflozin inhibits NHE1 activity in rabbit CM isolated in the absence of PXIV.
(A) Typical example of intracellular pH tracings for DMSO and EMPA-treated cells isolated without PXIV treatment in response to a NH_4^+ pulse; (B) Typical example of intracellular $[H^+]$ during the first 200 sec after NH_4^+ wash-out in control (DMSO) and EMPA-treated cells, showing the slope of the linear fit of the first 50 sec intracellular $[H^+]$ recovery; (C) NHE1 activity of control (DMSO) and EMPA-treated cells at an extracellular pH of 7.4, as determined by the slope of intracellular $[H^+]$ recovery in rabbit ventricular myocytes isolated without PXIV. (n=3/7 cells from 3/4 rabbits, Paired t test (two tailed)). EMPA: Empagliflozin. Slope ($\Delta[H^+]/\Delta s$) is the linear fit over first 50 s of intracellular $[H^+]$ recovery. * $P < 0.05$.

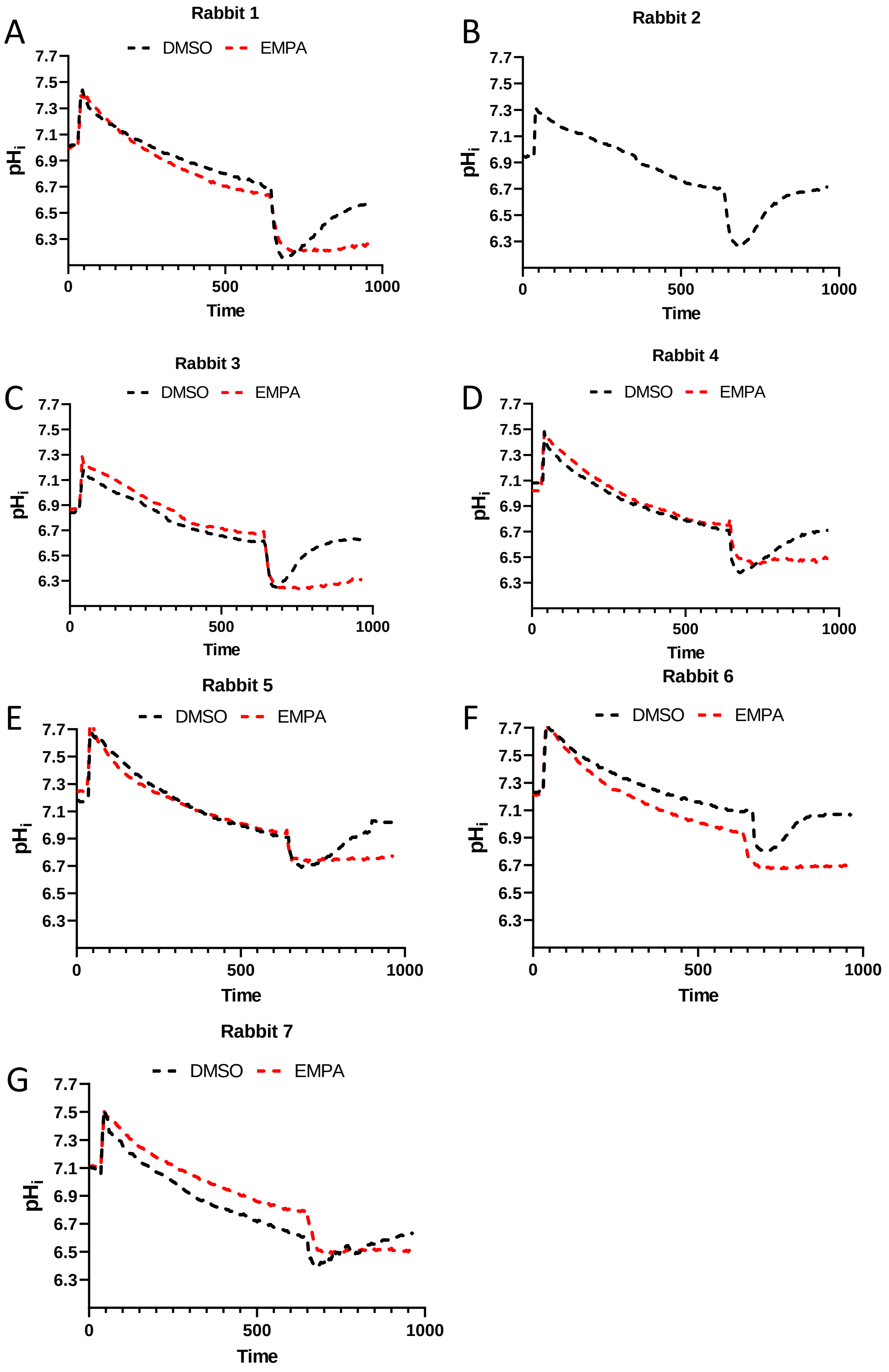

Supplementary figure 2. Empagliflozin inhibits NHE1 activity in rabbit CM isolated with 10 min PXIV perfusion. (A-G) Intracellular pH curves in response to a NH_4^+ pulse for DMSO- and EMPA-treated CMs isolated with 10 min PXIV during enzymatic dissociation using Langendorff perfusion for each of the 7 rabbits, respectively: rabbit1 (2/2 cells); rabbit2 (2/0 cells); rabbit3 (3/4 cells); rabbit4 (1/1 cells); rabbit5 (1/2 cells); rabbit6 (1/1 cells); rabbit7 (2/2 cells). EMPA: Empagliflozin.

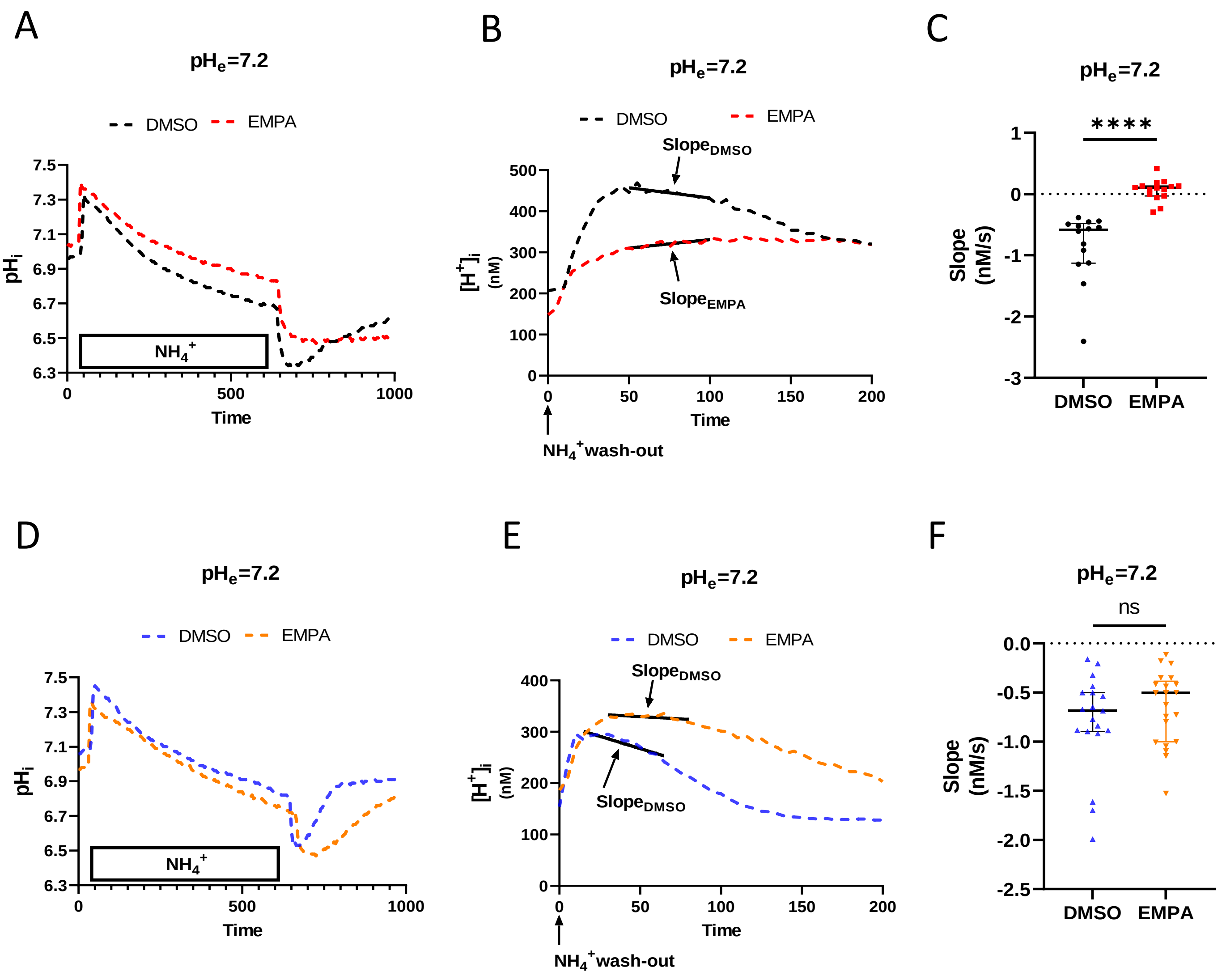

Supplementary figure 3. Empagliflozin does not inhibit NHE1 activity in rabbit CM obtained from 10 min PXIV perfusion + 20 min PXIV incubation. (A-C) Typical example of intracellular pH for DMSO and EMPA treatment after an ammonium pulse (A), $[H^+]$ and slope fitting (B) and summary data (C) for DMSO and EMPA-treated CM from 10 min PXIV perfusion at pH 7.2 (n=14/15 cells from 5 rabbits, Mann-Whitney test). (D-F) Typical example of intracellular pH (D), $[H^+]$ and slope fitting (E) and summary data (F) for DMSO and EMPA-treated CM obtained from 10 min PXIV perfusion + 20 min PXIV incubation at pH 7.2 (n=19/21 cells from 5 rabbits, Mann-Whitney test). EMPA: Empagliflozin. Slope ($\Delta[H^+]/\Delta s$) is the linear fit over first 50 s of intracellular $[H^+]$ recovery. ns $P > 0.05$, **** $P < 0.0001$.

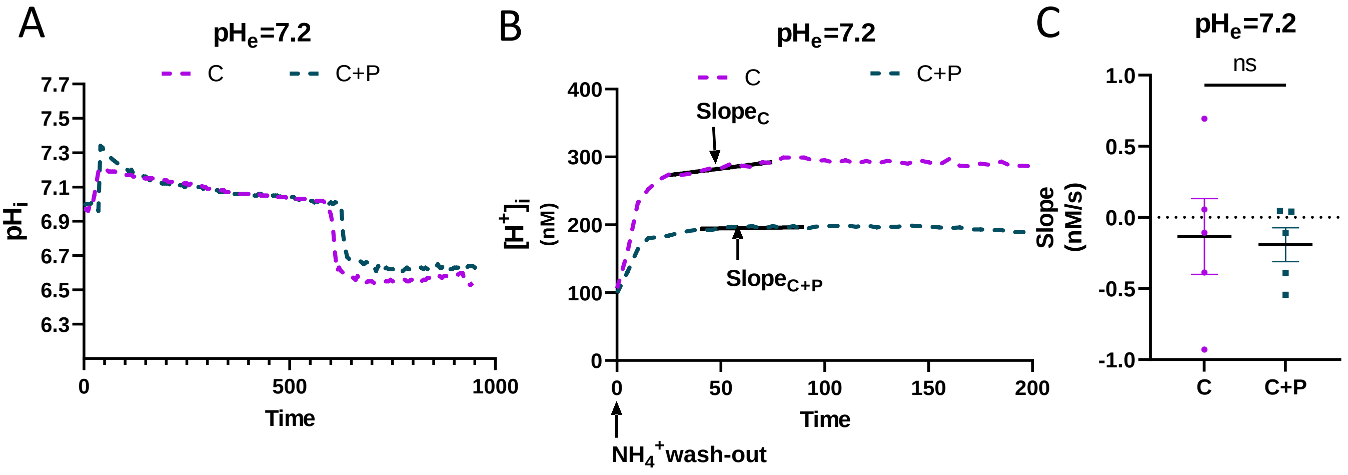

Supplementary figure 4. NHE1 inhibition by the NHE1 inhibitor cariporide is unaffected by PXIV treatment. (A-C) Average curve changes of intracellular pH (A), $[H^+]$ and slope fitting (B) and summary data (C) of NHE1 activity for H9c2 cells treated for 10 min with 10µM Cariporide with or without PXIV during $NH4^+$ pulse and the pH recovery period (n=5/5 cells from 5 independent experiments, Mann-Whitney test). C: Cariporide. C+P: Cariporide+ PXIV. Slope ($\Delta[H^+]/\Delta s$) is the linear fit over first 50 s of intracellular $[H^+]$ recovery. ns $P > 0.05$.
